# Dynamic Regulation of Quaternary Organization of the M_1_ Muscarinic Receptor by Subtype-selective Antagonist Drugs*

**DOI:** 10.1074/jbc.M115.712562

**Published:** 2016-04-14

**Authors:** John D. Pediani, Richard J. Ward, Antoine G. Godin, Sara Marsango, Graeme Milligan

**Affiliations:** From the ‡ Molecular Pharmacology Group, Institute of Molecular, Cell and Systems Biology, College of Medical, Veterinary and Life Sciences, University of Glasgow, Glasgow G12 8QQ, Scotland, United Kingdom and; § Institut d'Optique and CNRS, Laboratoire Photonique, Numérique et Nanosciences (LP2N) and Université de Bordeaux, LP2N, F-33405, UMR 5298, 33405 Talence Cedex, France

**Keywords:** 7-helix receptor, G protein-coupled receptor (GPCR), molecular pharmacology, oligomerization, receptor, signal transduction, transmembrane domain

## Abstract

Although rhodopsin-like G protein-coupled receptors can exist as both monomers and non-covalently associated dimers/oligomers, the steady-state proportion of each form and whether this is regulated by receptor ligands are unknown. Herein we address these topics for the M_1_ muscarinic acetylcholine receptor, a key molecular target for novel cognition enhancers, by using spatial intensity distribution analysis. This method can measure fluorescent particle concentration and assess oligomerization states of proteins within defined regions of living cells. Imaging and analysis of the basolateral surface of cells expressing some 50 molecules·μm^−2^ human muscarinic M_1_ receptor identified a ∼75:25 mixture of receptor monomers and dimers/oligomers. Both sustained and shorter term treatment with the selective M_1_ antagonist pirenzepine resulted in a large shift in the distribution of receptor species to favor the dimeric/oligomeric state. Although sustained treatment with pirenzepine also resulted in marked up-regulation of the receptor, simple mass action effects were not the basis for ligand-induced stabilization of receptor dimers/oligomers. The related antagonist telenzepine also produced stabilization and enrichment of the M_1_ receptor dimer population, but the receptor subtype non-selective antagonists atropine and *N*-methylscopolamine did not. In contrast, neither pirenzepine nor telenzepine altered the quaternary organization of the related M_3_ muscarinic receptor. These data provide unique insights into the selective capacity of receptor ligands to promote and/or stabilize receptor dimers/oligomers and demonstrate that the dynamics of ligand regulation of the quaternary organization of G protein-coupled receptors is markedly more complex than previously appreciated. This may have major implications for receptor function and behavior.

## Introduction

Encoded by single polypeptides that span the plasma membrane seven times and frequently considered to be monomeric entities, it is now well established that many class A, rhodopsin family, G protein-coupled receptors (GPCRs)^3^ can form dimers and/or oligomers when expressed in heterologous cell systems (1, 2). There is also growing evidence that the same may be true in native tissues (1, 2). Despite this there are many questions that remain unexplored or unresolved. These include the proportion of a receptor population that is present in such quaternary complexes, how this is affected by receptor expression level, and whether such complexes are regulated by interaction with ligands or other receptor modulators.

The family of muscarinic acetylcholine receptors, of which there are five subtypes in mammalian species (3), is a useful example to illustrate each of these issues. For example, studies on the muscarinic M_2_ receptor subtype have variously concluded that it may be predominantly monomeric but with a capacity to form dimers (4), is routinely dimeric (5), or is predominantly tetrameric (6). Similar variation has been reported for each of the muscarinic M_1_ (5, 7) and M_3_ (8–11) subtypes. In addition, although certain studies have indicated that addition of muscarinic ligands does not affect the prevalence of receptor dimers/oligomers (5, 12), other studies have indicated a capacity for regulation. For example, the presence of muscarinic M_2_ receptor homomers increased, whereas co-expressed M_2_/M_3_ heteromers concomitantly decreased in parallel, in an agonist-dependent manner, in cells co-expressing M_2_ and M_3_ receptors (13), and earlier studies had suggested that M_3_ receptor homomers also either reorganized or were increased in amount in response to agonist ligands (14). These latter studies were of particular interest because the extent of the agonist-mediated effect was greatest at lower receptor expression levels and less marked at higher receptor expression levels (14). This suggests that receptor expression level may indeed play a key role and that mass action might drive dimer/oligomer production at higher receptor expression levels.

**FIGURE 1.**
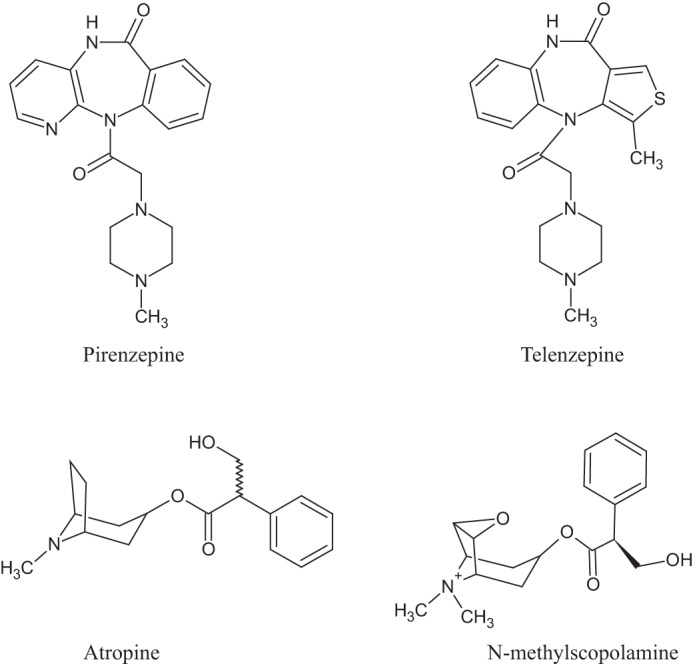
**Structures of muscarinic antagonists.** The chemical structures of pirenzepine, telenzepine, atropine, and *N*-methylscopolamine are shown.

Pirenzepine (11-[(4-methylpiperazin-1-yl)acetyl]-5,11-dihydro-6*H*-pyrido[2,3-*b*][1,4]benzodiazepin-6-one) (Fig. 1) is a particularly interesting ligand in the history of muscarinic receptor pharmacology because it was the first antagonist shown to have substantially different affinity at muscarinic receptors in distinct tissues and in different regions of the central nervous system (CNS). It was thus integral in defining that there must be more than one subtype of muscarinic acetylcholine receptor (15), an idea subsequently confirmed by cloning of the distinct receptor subtypes (3). Pirenzepine displays substantially higher affinity for M_1_ than M_2_ receptors. Pirenzepine is also a medicine clinically used to treat gastric ulcers as is the closely related molecule telenzepine (4,9-dihydro-3-methyl-4-[4-methyl-1-piperazinyl)acetyl]-10*H*-thieno[3,4-*b*][1,5]benzodiazepin-10-one) (Fig. 1). These are useful medicines because their marked selectivity for the M_1_ receptor subtype means that they do not significantly increase heart rate unlike the subtype non-selective, antimuscarinic drugs including atropine and *N*-methylscopolamine (Fig. 1) as this is an M_2_ subtype-mediated response. Moreover, as they do not cross the blood-brain barrier effectively, they also do not inhibit M_1_-mediated cholinergic function in the CNS.

We have been exploring various approaches to define transmembrane protein organization and potential reorganization (16, 17). Herein we examine effects of pirenzepine on human M_1_ receptor quaternary organization by using confocal fluorescence imaging and then subjecting such images to spatial intensity distribution analysis (SpIDA) (18–21). We show that treatment of cells induced to express the human M_1_ receptor with pirenzepine dramatically increases the proportion of dimeric/oligomeric forms and that this is reversed following removal of the ligand. We also show that pirenzepine-induced dimerization is independent of a concomitant, marked increase in cell surface level of the receptor that is produced with sustained exposure to this ligand. Telenzepine, a closely related ligand that has higher affinity for the M_1_ receptor than pirenzepine, produced equivalent results, but interestingly the standard subtype non-selective antagonists atropine and *N*-methylscopolamine did not. Moreover, although pirenzepine and telenzepine can also bind to the M_3_ muscarinic receptor, these ligands did not produce such effects at the M_3_ receptor even when used at concentrations that fully occupy this receptor subtype.

## Experimental Procedures

### 

#### 

##### Materials

General laboratory chemicals as well as atropine ((*RS*)-(8-methyl-8-azabicyclo[3.2.1]oct-3-yl) 3-hydroxy-2-phenylpropanoate), *N*-methylscopolamine (NMS), cytochalasin D, and both anti-tubulin antibody and secondary horseradish peroxidase-conjugated antibody were from Sigma-Aldrich or Fisher Scientific. DNA restriction endonucleases, calf intestinal alkaline phosphatase, T4 DNA polymerase, and T4 ligase were from New England Biolabs (Hitchin, UK). Wizard Plus SV Miniprep kit was from Promega (Southampton, UK). NuPAGE Novex precast 4–12% Bis-Tris gels and NuPAGE MOPS SDS running buffer were from Invitrogen. QIAfilter Plasmid Maxi kit, PCR purification kit, and QIAquick gel extraction kit were from Qiagen (Crawley, UK). Agarose was from Flowgen Biosciences (Nottingham, UK). The anti-GFP antiserum was generated in-house. ECL reagent was purchased from Pierce. [^3^H]Quinuclidinylbenzilate ([^3^H]QNB) and [*N-methyl*-^3^H]scopolamine methyl chloride ([^3^H]NMS) were from PerkinElmer Life Sciences. Other muscarinic ligands, specifically pirenzepine and telenzepine were from Tocris (Bristol, UK). Hanks' balanced salt solution was from Life Technologies.

##### DNA Constructs

Enhanced green fluorescent protein incorporating an A206K mutation to reduce any tendency for the fluorescent protein to homodimerize (monomeric enhanced green fluorescent protein (mEGFP)) (22) was a gift from Dr. K. Herrick-Davis (Albany, NY). To localize mEGFP to the plasma membrane, a palmitoylation-myristoylation (P-M) sequence (23) was added to the N terminus of the fluorescent protein as described (24). hM_1_-mEGFP was also a gift from Dr. K. Herrick-Davis. hM_3_-mEGFP was made by subcloning PCR-amplified hM_3_ between the SacI and BamHI sites of the mEGFP vector. To allow the generation of inducible Flp-In^TM^ T-REx^TM^ stable cell lines, these constructs were all transferred from the pEGFPN-1 plasmid (Takara Bio Europe/Clontech) backbone to pcDNA5-FRT-TO (Invitrogen). This was done by excising the appropriate region of the plasmid with NheI and NotI and subcloning into the pcDNA5/FRT/TO vector at EcoRV-NotI (after blunting the NheI site). All constructs were verified by sequencing.

##### Cell Lines

All cells were maintained in a humidified incubator with 95% air and 5% CO_2_ at 37 °C. Parental Flp-In T-REx 293 cells (Invitrogen) were maintained in DMEM (high glucose) supplemented with 10% (v/v) fetal calf serum, 100 units·ml^−1^ penicillin, 0.1 mg·ml^−1^ streptomycin, 10 μg·ml^−1^blasticidin, and 100 μg·ml^−1^ Zeocin. Cell lines generated from Flp-In T-REx 293 parental cells were maintained in DMEM (high glucose) supplemented with 10% (v/v) fetal calf serum, 100 units·ml^−1^ penicillin, 0.1 mg·ml^−1^ streptomycin, 10 μg·ml^−1^ blasticidin, and 200 μg·ml^−1^ hygromycin.

##### Stable Cell Line Generation

Inducible Flp-In T-REx stable cell lines able to express P-M-mEGFP, hM_1_-mEGFP, or hM_3_-mEGFP were generated as described (24). After 48 h, the medium was changed to medium supplemented with 200 μg·ml^−1^ hygromycin to initiate selection of stably transfected cells. Pools of cells were established (10–14 days for resistant colonies to form) and tested for inducible expression by the addition of 0.1 μg·ml^−1^ doxycycline for 48 h followed by screening for fluorescence corresponding to EGFP or by immunoblotting.

##### Cell Treatments

For antagonist treatments, cells were incubated with the appropriate concentration of compound for 16 h at 37 °C. For cytoskeletal disruption studies, cells were incubated with 2.5 μg·ml^−1^ cytochalasin D for 3 h at 37 °C.

##### Generation of Cell Lysates and Immunoblotting

Cells were washed once in cold phosphate-buffered saline (PBS) (120 mm NaCl, 25 mm KCl, 10 mm Na_2_HPO_4_, and 3 mm KH_2_PO_4_, pH7.4) and harvested with ice-cold radioimmunoprecipitation assay buffer (50 mm HEPES, 150 mm NaCl, 1% Triton X-100, 0.5% sodium deoxycholate, 10 mm NaF, 5 mm EDTA, 10 mm NaH_2_PO_4_, and 5% ethylene glycol, pH 7.4) supplemented with Complete protease inhibitor mixture (Roche Diagnostics). Extracts were passed through a 25-gauge needle and incubated for 15 min at 4 °C while on a rotating wheel. Cellular extracts were then centrifuged for 10 min at 21,000 × *g*, and the supernatant was recovered. Samples were prepared by the addition of sodium dodecyl sulfate-polyacrylamide gel electrophoresis (SDS-PAGE) sample buffer and heated to 65 °C for 5 min before being subjected to SDS-PAGE analysis using 4–12% Bis-Tris gels (NuPAGE; Invitrogen) and MOPS buffer. After separation, the proteins were electrophoretically transferred to nitrocellulose membrane, which was then blocked (5% fat-free milk powder in PBS with 0.1% Tween 20) at 4 °C on a rotating shaker overnight. The membrane was incubated for 3 h with primary antibody (1:10,000 sheep anti-GFP) in 2% fat-free milk powder in PBS-Tween, washed (3 × 10 min with PBS-Tween), and then incubated for 3 h with appropriate secondary antibody (horseradish peroxidase-linked rabbit anti-goat IgG diluted 1:10,000 in 2% fat-free milk powder in PBS-Tween). After washing as above, proteins were detected by enhanced chemiluminescence (Pierce Chemical) according to the manufacturer's instructions.

##### Cell Membrane Preparations

Cells induced with the required concentration of doxycycline to express hM_1_-mEGFP or hM_3_-mEGFP were washed and then harvested with ice-cold PBS. Pellets of cells were frozen at −80 °C for a minimum of 1 h, thawed, and resuspended in ice-cold 10 mm Tris and 0.1 mm EDTA, pH 7.4 (TE buffer) supplemented with Complete protease inhibitor mixture. Cells were homogenized on ice by 40 strokes of a glass-on-Teflon homogenizer followed by centrifugation at 1000 × *g* for 5 min at 4 °C to remove unbroken cells and nuclei. The supernatant fraction was removed and passed through a 25-gauge needle 10 times before being transferred to ultracentrifuge tubes and subjected to centrifugation at 90,000 × *g* for 30 min at 4 °C. The resulting pellets were resuspended in ice-cold TE buffer. Protein concentration was assessed, and membranes were stored at −80 °C until required.

##### [^3^H]QNB Binding Assays

Both single concentration binding studies and saturation binding curves were established by the addition of 20 μg of membrane protein to assay buffer (20 mm HEPES, 100 mm NaCl, and 10 mm MgCl_2_, pH 7.5) containing either a single, near saturating concentration (5 nm), or varying concentrations of [^3^H]QNB (0.01–30 nm). Nonspecific binding was determined in the presence of 10 μm atropine. Reactions were incubated for 120 min at 30 °C, and bound ligand was separated from free by vacuum filtration through GF/C filters (Brandel Inc., Gaithersburg, MD) that had been presoaked in assay buffer. The filters were washed twice with cold assay buffer, and bound ligand was estimated by liquid scintillation spectrometry. Competition binding assays were carried out in a similar way but with a constant concentration of [^3^H]QNB (1 nm) and the addition of a range of concentrations of ligands of interest (0.03 nm–1 mm). Data were analyzed using GraphPad Prism 5.03 (GraphPad Software, La Jolla, CA).

##### [^3^H]NMS Binding Assay

Flp-In T-REx 293 cells able to express a construct of interest were grown overnight on white 96-well microtiter plates that had been treated with 0.1 mg·ml^−1^ poly-d-lysine. Cells were then treated with various concentrations of doxycycline for 24 h at 37 °C. The medium was removed and replaced with 100 μl/well cold PBS containing 1 nm [^3^H]NMS. Nonspecific binding was determined in the presence of 10 μm atropine. The plates were incubated at 4 °C for 150 min, and the assay was terminated by removal of the binding mixture followed by washing with 4 × 100 μl/well ice-cold PBS. One hundred microliters/well Microscint 20 (PerkinElmer Life Sciences) was added, and the plates were sealed before overnight incubation at room temperature on a rapidly shaking platform. Bound ligand was determined using a Packard Topcount NXT (PerkinElmer Life Sciences). Using the specific binding per well and number of cells per well, the receptor copies per cell was determined.

##### Inositol Monophosphate Assay

Inositol monophosphate accumulation assays were performed using Flp-In T-REx 293 cells able to express the hM_3_-mEGFP receptor construct in an inducible manner. Experiments were performed using a homogenous time-resolved FRET-based detection kit (CisBio Bioassays, Codolet, France) according to the manufacturer's protocol. Cells were plated at 7500 cells/well in low volume 384-well plates, and the ability of various concentrations of the agonist carbachol to increase the level of inositol monophosphate was assessed following incubation for 2 h with the agonist. In appropriate experiments, this was preceded by a 15-min preincubation with the indicated concentrations of antagonist (atropine, pirenzepine, or telenzepine).

##### Monitoring of mEGFP Fluorescence Emission Spectrum

Flp-In T-REx 293 cell lines able to express hM_1_-mEGFP were grown to 100,000 cells/well in 96-well solid black bottom plates (Greiner Bio-One) precoated with 0.1 mg·ml^−1^ poly-d-lysine. Cells were treated with 100 ng·ml^−1^ doxycycline to induce the expression of hM_1_-mEGFP. After 24-h induction, cells were washed three times in Hanks' balanced salt solution buffer. 100 μl of Hanks' balanced salt solution was added to each well, and the plates were read using a CLARIOstar fluorescence plate reader (BMG Labtechnologies). Specifically, cells were excited at 462 nm, and the emission spectrum between 500 and 600 nm was collected at 5-nm intervals. The same process was repeated after the addition to each well of 100 μl of Hanks' balanced salt solution supplemented with the vehicle or the appropriate muscarinic receptor antagonist.

##### SpIDA

SpIDA was carried out essentially as described (24). All region of interest (RoI) measurements were selected from the basolateral membrane surface. Monomeric equivalent unit (MEU) values for hM_1_-mEGFP or hM_3_-mEGFP were measured by normalizing their quantified quantal brightness (QB) values to average QB values measured from the P-M-mEGFP construct using exactly the same laser power as used to excite the muscarinic receptor subtype constructs. To distinguish between monomeric and dimeric/oligomeric hM_1_-mEGFP or hM_3_-mEGFP species, P-M-mEGFP MEU occurrence/frequency *x-y* graphs (MEU bin size = 0.1) were plotted for each MEU value measured during excitation with laser power set to 2 or 6%. Such plots revealed a symmetrical distribution of the values, and GraphPad Prism normality tests indicated the distributions were Gaussian (see “Results” and “Statistical Analyses”). The data from each frequency *x-y* plot measured using 2 or 6% laser power were combined as this range of excitation settings was required to illuminate the muscarinic receptor subtypes optimally at differing expression levels without erroneous detector pixel saturation occurring. From this combined plot, an MEU value of 1.274 (which represented 75% of the data set, falling within the mean ± 1.5 standard deviations) was set as a border to distinguish between monomeric and larger complexes in studies where individual MEU values exceeded 1.274. The distribution of such values for the muscarinic receptor constructs was non-Gaussian and skewed toward higher values.

##### Calculation of Receptor Density at the Cell Surface by SpIDA

SpIDA software also reports the mean fluorescence intensity for each RoI analyzed. The number of hM_1_-mEGFP, hM_3_-mEGFP, or P-M-mEGFP molecules/μm^−2^ (density) was measured by dividing the mean fluorescence intensity value by the quantified monomeric QB value.

##### Statistical Analyses

Variation in receptor number or mean/median of QB produced by treatment with either ligands or varying concentrations of doxycycline was assessed by one-way analysis of variance with the use of Bonferroni's or Dunnett's test for multiple comparisons as appropriate. Normality distributions of recovered QB values defined as MEUs were assessed by each of D'Agostino and Pearson, Kolmogorov-Smirnov, and Shapiro and Wilk normality tests (at *p* > 0.05) and by skewness and Kurtosis assessments. Distributions that failed any of the three normality assessments (at *p* < 0.05) were considered to be non-Gaussian.

## Results

**FIGURE 2.**
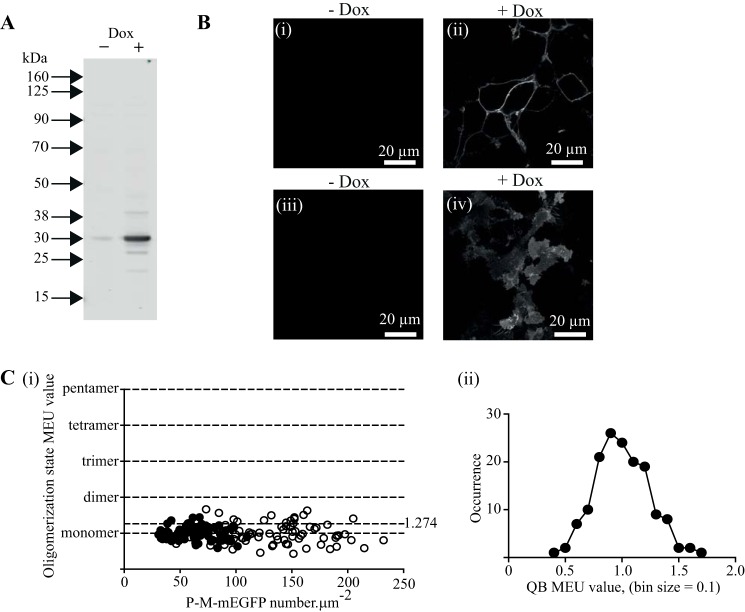
**Expression, cellular distribution profile, and quantal brightness analysis of plasma membrane-targeted mEGFP.** Flp-In T-REx 293 cells harboring P-M-mEGFP were maintained in the absence of doxycycline (− *Dox*) or treated with doxycycline (10 ng·ml^−1^) for 24 h (+ *Dox*). Lysates of these cells were resolved by SDS-PAGE and immunoblotted with an anti-GFP antiserum (*A*). In *B*, images of uninduced cells (*panels i* and *iii*) or cells induced to express P-M-mEGFP (*panels ii* and *iv*) are shown. *B*, *panels i* and *ii*, confocal images across groups of cells. *B*, *panels iii* and *iv*, images of the basolateral surface of such cells. *C*, *panel i*, shows QB assessed in individual RoIs (presented as monomeric equivalent units) plotted against number of mEGFP·μm^−2^ of the basolateral surface. *Filled symbols*, cells treated with 2.5 ng·ml^−1^ doxycycline; *open symbols*, cells treated with 10 ng·ml^−1^ doxycycline. *C*, *panel ii*, QB values from individual RoIs were binned (bin size, 0.1 MEU), and these displayed a symmetrical distribution (see “Results”).

A cDNA encoding A206K mEGFP that incorporated an N-terminal addition of a plasma membrane-targeting P-M sequence was cloned into the doxycycline-inducible Flp-In T-REx locus of Flp-In T-REx 293 cells. Following addition of doxycycline (10 ng·ml^−1^) to these cells, a polypeptide of the anticipated molecular mass (30 kDa) was identified by immunoblotting SDS-PAGE-resolved cell lysates with an anti-GFP antiserum (Fig. 2*A*). Confocal imaging of the cells confirmed plasma membrane targeting of the P-M-mEGFP construct following addition of doxycycline (Fig. 2*B*, *panels i* and *ii*). Imaging RoIs of the basolateral membrane of these cells (Fig. 2*B*, *panels iii* and *iv*) and subsequent analysis of such images by SpIDA (18, 19, 21, 24, 25) indicated that, with laser power set to 2%, this polypeptide displayed QB of 12.15 ± 0.39 units (mean ± S.E., *n* = 76). Based on fluorescence intensity measurements, in this set of studies the average expression level of P-M-mEGFP within the basolateral membrane was 133.8 ± 4.7 molecules·μm^−2^ (mean ± S.E., *n* = 76) and in individual RoIs this ranged between 46 and 232 molecules·μm^−2^ (Fig. 2*C*, *panel i*). Variation in the concentration of doxycycline used can allow control of the level of expression of the polypeptide harbored at the Flp-In T-REx locus of such cells. Following treatment of these cells with 2.5 ng·ml^−1^ doxycycline, levels of expression of P-M-mEGFP were lower, and appropriate analysis of confocal images taken from the cells required laser power to be increased. With laser power set to 6%, the P-M-mEGFP polypeptide displayed QB of 25.24 ± 0.54 (mean ± S.E., *n* = 76) with an average expression level of 62.1 ± 2.2 molecules·μm^−2^. In individual RoIs, this ranged between 1 and 109 molecules·μm^−2^ (Fig. 2*C*, *panel i*).

To assess whether P-M-mEGFP was detected as monomeric across the full expression range achieved, we combined these two data sets. This resulted in an average expression level of 99.0 ± 3.9 molecules·μm^−2^ (mean ± S.E., *n* = 152). Information from each individual RoI was plotted as multiples of the average QB, *i.e.* 12.15 or 25.24, respectively, for those obtained with laser power set to 2 or 6%, and defined as MEU (Fig. 2*C*, *panel i*). Analysis of this combined data set showed it to be consistent with Gaussian distribution, and as such there was no evidence to suggest that at higher levels of expression proximity of individual molecules of P-M-mEGFP resulted in incorrect assignment of these as being non-monomeric (Fig. 2*C*, *panel ii*). Based on the distribution of values of MEU for P-M-linked mEGFP that represented 75% of the data set, falling within the mean ± 1.5 S.D., in subsequent studies we defined QB values ≤1.274 MEU as being monomeric, whereas QB values >1.274 MEU were regarded as reflecting complexes that were larger than monomers (Fig. 2*C*, *panel ii*) (see “Experimental Procedures” for further details).

**FIGURE 3.**
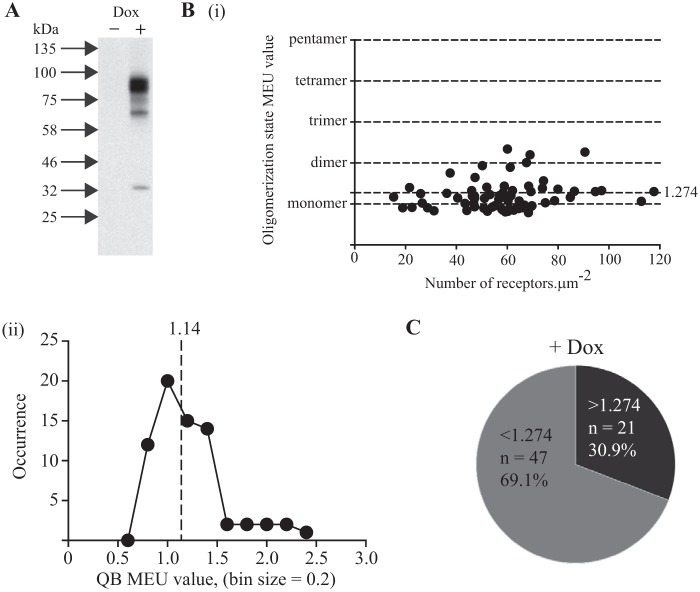
**hM_1_-mEGFP exists as a mixture of monomers and oligomers in the basal state.** Flp-In T-REx 293 cells harboring hM_1_-mEGFP were maintained in the absence of doxycycline (− *Dox*) or treated with doxycycline (100 ng·ml^−1^) for 24 h (+ *Dox*). Lysates of these cells were resolved by SDS-PAGE and immunoblotted with an anti-GFP antiserum (*A*). *B*, *panel i*, shows QB detected in individual RoIs (presented as monomeric equivalent units) plotted against number of hM_1_-mEGFP·μm^−2^ of the basolateral surface (55.7 ± 2.3 μm^−2^, mean ± S.E., *n* = 68). *B*, *panel ii*, QB values from individual RoIs were binned (bin size, 0.2 MEU). These displayed a non-symmetrical distribution skewed to values >1.00 MEU. *Dotted line*, median value. *C*, in this data set, 47 of the 68 measurements were assessed as being predominantly monomeric (69.1%).

We next generated equivalent Flp-In T-REx 293 cells in which cDNA encoding a form of the human M_1_ muscarinic receptor with C-terminally fused mEGFP (hM_1_-mEGFP) was cloned into the Flp-In T-REx locus. Once more this allowed expression of the receptor construct only upon addition of doxycycline with maximal expression obtained by use of 100 ng·ml^−1^ doxycycline. The receptor was detected as a single species, of apparent molecular mass close to 80 kDa, by immunoblotting lysates of untreated and doxycycline-treated cells following resolution by SDS-PAGE (Fig. 3*A*). Fluorescence intensity measurements of RoI from the basolateral membrane of these cells indicated hM_1_-mEGFP to be expressed at 58.3 ± 2.4 copies·μm^−2^ (*n* = 68) but with extremes of variation across these RoIs of greater than 4-fold (Fig. 3*B*, *panel i*). SpIDA of the images from these RoI, assessed at 2% laser power, indicated a median QB of 13.85 units (*n* = 68). This corresponds to 1.14 MEU (*i.e.* 1.14 MEU times the mean value of the monomeric P-M-linked mEGFP). Here, however, distribution of the individual observations was non-Gaussian with skew toward higher values (Fig. 3*B*, *panel ii*). This is consistent with, at this expression level, the majority of the receptor construct being monomeric but with a proportion being within larger quaternary structures, *i.e.* dimeric and/or oligomeric. The percentage of hM_1_-mEGFP RoI QB values defined as “larger than monomer” in this data set was 30.9% (Fig. 3*C*). Although very modest (*r*^2^ = 0.032), over this limited range of expression levels, there was a positive correlation between receptor number and the presence of non-monomeric species observed in distinct RoIs (see later). Although the number of observations in this specific data set was restricted to 68, subsequently, at the completion of the experimental studies, we combined the full data sets generated for cells induced to express hM_1_-mEGFP but not further treated (*n* = 478). Analysis confirmed the non-Gaussian distribution of the QB corresponding to hM_1_-mEGFP and allowed estimation of the percentage of RoI QB values that were consistent with the mean basal receptor being larger than monomer as 25.7%.

**FIGURE 4.**
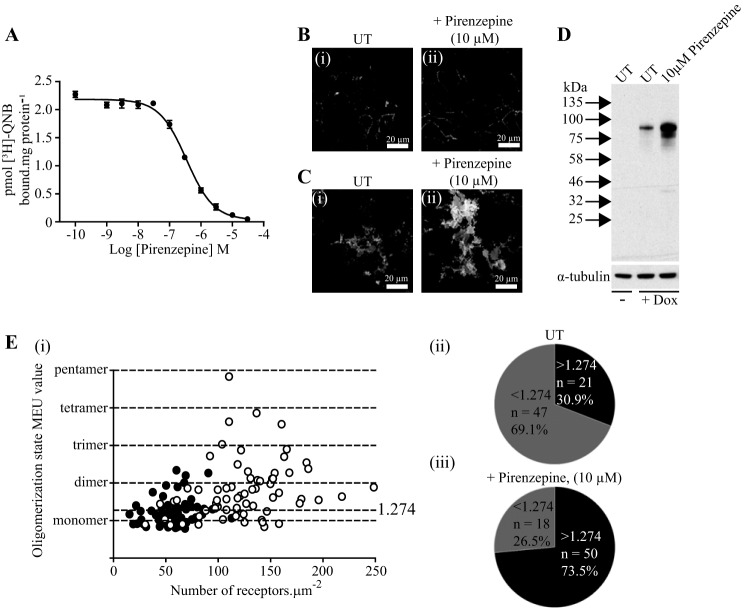
**Sustained treatment with pirenzepine causes relocation and up-regulation of hM_1_-mEGFP and promotes oligomerization of the receptor.** Flp-In T-REx 293 cells harboring hM_1_-mEGFP were treated with doxycycline (100 ng·ml^−1^) for 24 h. *A*, membrane preparations from these cells were used in ligand binding studies using 1 nm [^3^H]QNB and varying concentrations of pirenzepine. Based on the *K_d_* for [^3^H]QNB, estimated as 98 ± 17 pm, the inhibition constant (p*K_i_*) for pirenzepine was calculated as 7.66 ± 0.04. Shown is a representative graph from *n* = 3; *error bars* represent ±S.E. Cells were then treated with vehicle (*UT*) or with pirenzepine (10 μm) for 24 h. *B*, *panels i* and *ii*, confocal images as in Fig. 2 showed that treatment with pirenzepine resulted in relocation of hM_1_-mEGFP to enhance cell surface/plasma membrane localization. *C*, *panels i* and *ii*, imaging the basolateral surface of a group of cells indicated that pirenzepine caused up-regulation of hM_1_-mEGFP. *D*, immunoblotting studies confirmed that pirenzepine caused up-regulation of hM_1_-mEGFP. α-Tubulin acted as a loading control. *E*, *panels i–iii*, quantal brightness and fluorescence intensity analysis from SpIDA confirmed an overall 2.1-fold up-regulation of receptor number at the basolateral surface and that a substantially greater proportion of the RoIs were identified as containing dominantly receptor dimers/oligomers (73.5%) following pirenzepine treatment (*open symbols*) than in vehicle-treated controls (30.9%) (*filled symbols*).

**FIGURE 5.**
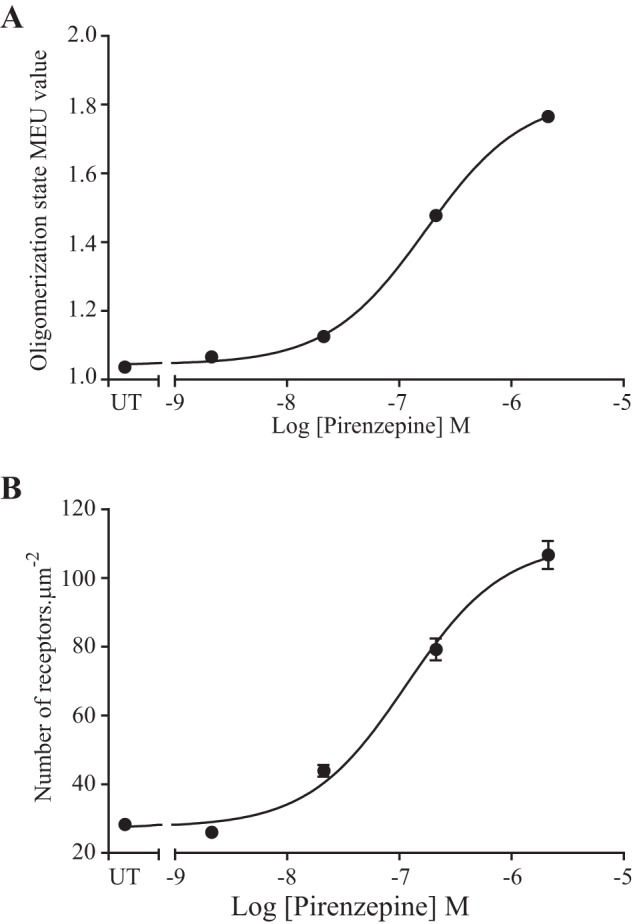
**Pirenzepine-induced increases in both receptor oligomerization and expression levels are concentration-dependent.** Experiments akin to those of Fig. 4 were performed on Flp-In T-REx 293 cells harboring hM_1_-mEGFP that had been treated with doxycycline (100 ng·ml^−1^) for 24 h and then treated with the indicated concentrations of pirenzepine. QB values corresponding to hM_1_-mEGFP were plotted as monomeric equivalent units against pirenzepine concentration (*A*) as were the number of receptor molecules·μm^−2^ (*B*). Data are presented as median (*A*) and mean (*B*). *Error bars* represent ±S.E.; *n* = 84 in each case. *UT*, untreated.

Pirenzepine (15) is the prototypic M_1_-selective muscarinic receptor antagonist and has been reported to stabilize dimers of this receptor (26). Competition binding experiments between pirenzepine and the muscarinic antagonist [^3^H]QNB (*K_d_* = 98 ± 17 pm) allowed definition of the affinity of pirenzepine (p*K_i_* = 7.66 ± 0.04) for the hM_1_-mEGFP construct (Fig. 4*A*). Initially Flp-In T-REx 293 cells induced to express hM_1_-mEGFP by treatment with 100 ng·ml^−1^ doxycycline were incubated for 16 h with vehicle or with a single concentration of pirenzepine (10 μm) calculated to be sufficient to occupy greater than 99% of the receptor population. Imaging of these vehicle- and pirenzepine-treated cells illustrated a number of features. First, confocal images across the center of the cells showed that, as with many GPCRs, although a substantial proportion of the hM_1_ receptor construct was located at the plasma membrane in vehicle-treated cells, a significant fraction was inside the cells and located within punctate vesicles (Fig. 4*B*, *panel i*). By contrast, following treatment with pirenzepine very little of the receptor was detected within the cells, and the construct was highly concentrated at the cell surface (Fig. 4*B*, *panel ii*). Imaging of the basolateral surface of groups of these cells indicated that there was marked up-regulation of hM_1_-mEGFP following such sustained treatment with pirenzepine (Fig. 4*C*), and this was confirmed in immunoblotting studies (Fig. 4*D*). Fluorescence intensity of RoI and associated SpIDA within the basolateral membrane of such pirenzepine-treated cells indicated that at 2% laser power the median QB of the receptor construct (19.50 units, *n* = 68, *i.e.* 1.61 MEU) increased markedly (*p* < 0.001) compared with the untreated cells (Fig. 4*E*). This was consistent with a substantial increase (to 73.5%) in the percentage of RoI containing predominantly dimeric/oligomeric complexes (Fig. 4*E*). Indeed, examination of the individual observations from distinct RoIs indicated a significant proportion of these to be potentially consistent with the receptor existing in complexes that were larger than dimers (Fig. 4*E*). Notably, the effect of pirenzepine to both enhance QB of hM_1_-mEGFP (Fig. 5*A*) and increase the number of copies of the receptor construct (Fig. 5*B*) was concentration-dependent with half-maximal effect produced by close to 100 nm ligand.

**FIGURE 6.**
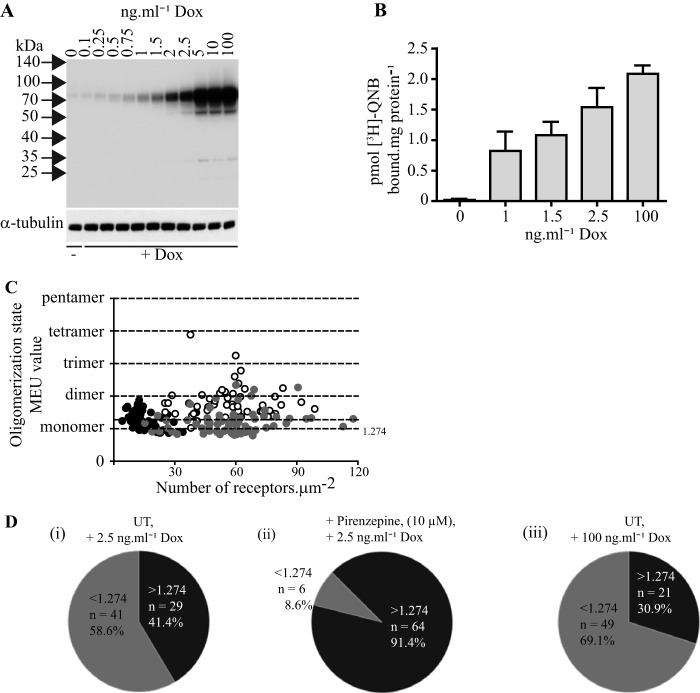
**Pirenzepine stabilizes hM_1_-mEGFP dimers/oligomers independently from effects on receptor expression levels.** Flp-In T-REx 293 cells harboring hM_1_-mEGFP were treated with varying concentrations of doxycycline (*Dox*). *A*, lysates from these cells were immunoblotted with an anti-GFP antiserum to assess relative expression levels of the receptor construct. α-Tubulin acted as a loading control. *B*, specific binding of a single near receptor saturating concentration of [^3^H]QNB (5 nm) was assessed in cell membrane preparations generated from cells treated with the indicated concentrations of doxycycline. Shown are combined data from *n* = 3 experiments; *error bars* represent ±S.E. *C*, comparisons of QB (presented as monomeric equivalent units) and receptor expression levels in cells induced to express hM_1_-mEGFP by treatment with 2.5 ng·ml^−1^ doxycycline (*black symbols*), treatment with 100 ng·ml^−1^ doxycycline (*gray symbols*), and after induction with 2.5 ng·ml^−1^ and pirenzepine treatment (*open symbols*). *D*, in this data set, 91% of the RoIs contained a majority of receptors present in dimeric/oligomeric form following treatment with pirenzepine (*panel ii*) but only 31 (*panel iii*) and 41% (*panel i*) in the absence of such ligand treatment. *UT*, untreated.

Although the oligomeric organization of hM_1_-mEGFP was clearly more complex after sustained treatment of the cells with pirenzepine, the correlation between receptor expression levels and receptor quaternary complexity was now also markedly increased (*r*^2^ = 0.22) compared with the untreated cells. However, we have shown previously that dimeric and oligomeric organization of another class A GPCR, the serotonin 5-HT_2C_ receptor, increases simply with expression level of the receptor (24). Therefore, because basolateral membrane receptor number was increased on average to 122.4 ± 5.1 copies·μm^−2^ following sustained pirenzepine treatment (Fig. 4*D*), it was impossible from these initial studies to determine whether pirenzepine directly promoted and/or stabilized dimeric/oligomeric forms of the hM_1_ receptor or whether receptor up-regulation produced at the basolateral surface of the cells by treatment with this antagonist ligand was sufficient by itself to account for these observations. A specific feature of the Flp-In T-REx locus is that the level of expression of polypeptides can be controlled by varying the concentration of doxycycline used (27). We, therefore, treated cells harboring hM_1_-mEGFP at this locus with a range of concentrations of doxycycline for 24 h. Immunoblotting SDS-PAGE-resolved lysates from these cells with the anti-GFP antiserum showed variation in expression of the receptor over the range 1–100 ng·ml^−1^ doxycycline (Fig. 6*A*). In parallel, binding of a near saturating concentration of [^3^H]QNB to membrane preparations of these cells provided confirmation that the increasing immunological identification of the receptor with doxycycline concentration was indeed consistent with increasing numbers of receptors that could bind the muscarinic antagonist (Fig. 6*B*). Based on these results, we selected to study organization of hM_1_-mEGFP in cells treated with 2.5 ng·ml^−1^ doxycycline. In such cells, analysis of basolateral RoIs confirmed that receptor expression was reduced (14.4 ± 0.7 receptors·μm^−2^) compared with those treated with the maximally effective concentration of doxycycline (Fig. 6*C*). Although this level was significantly lower (*p* < 0.001) than the 58.3 ± 2.4 copies·μm^−2^ recorded earlier in cells treated with 100 ng·ml^−1^ doxycycline, this did not result in a significant alteration in average QB and indicated that in both situations the majority of the receptors were monomeric (Fig. 6*C*). Now, although overnight treatment with pirenzepine (10 μm) once more significantly increased (*p* < 0.001) receptor density (now to 54.5 ± 1.9 copies·μm^−2^, *n* = 70) at the basolateral membrane (Fig. 6*C*), it did so only to the same levels as present in cells induced with 100 ng·ml^−1^ doxycycline but not treated with pirenzepine (*i.e.* 58.3 ± 2.4 copies·μm^−2^). QB and SpIDA assessment now indicated that, at the same level of basolateral cell surface expression of hM_1_-mEGFP, pirenzepine treatment had indeed produced a substantial increase in overall receptor complex organization as MEU increased to 1.73 (median). In these experiments, following pirenzepine treatment the vast majority of the RoIs (91.4%) had a higher average receptor oligomerization state than the monomer (*e.g.* dimeric/oligomeric), whereas without pirenzepine treatment only in 30% of the RoIs were there receptors that were not largely monomeric (Fig. 6*D*).

**FIGURE 7.**
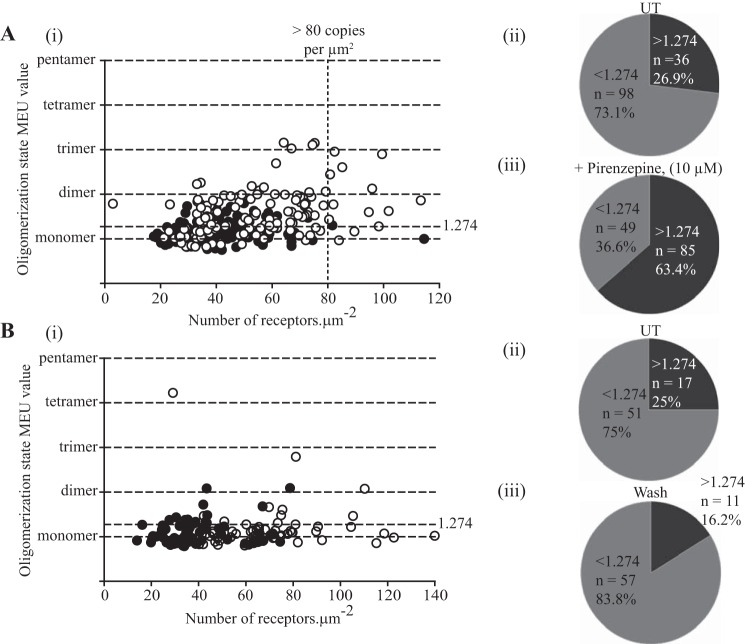
**Short term treatment with pirenzepine also promotes hM_1_ receptor dimer/oligomeric organization, and removal of the ligand results in reversal.** Flp-In T-REx 293 cells harboring hM_1_-mEGFP were treated with 100 ng·ml^−1^ doxycycline for 24 h to induce expression of the receptor construct. *A*, cells were then treated with pirenzepine (10 μm) (*open symbols*) or vehicle (*filled symbols*) for 90 min. Pirenzepine treatment resulted in a small increase in cell surface receptor levels (54.1 ± 1.7 *versus* 41.8 ± 1.3 copies·μm^−2^) (*panel i*) but a substantial increase in RoIs containing predominantly high order oligomers (63 *versus* 27%) (*panels ii* and *iii*). *B*, following washing of cells that had been exposed to pirenzepine (*open symbols*) or untreated (*filled symbols*), quaternary organization was assessed 90 min later (*panel i*). In this study, 25% of RoIs from the previously untreated cells were now estimated to have hM_1_-mEGFP in predominantly dimeric/oligomeric forms, whereas for those previously exposed to pirenzepine this was 16% (*panels ii* and *iii*). *UT*, untreated.

We next considered whether shorter term treatment with pirenzepine would also result in receptor dimerization/oligomerization without causing the potentially confounding degree of receptor up-regulation. For these studies, we used cells that in the absence of pirenzepine had been induced to express the receptor by treatment with 100 ng·ml^−1^ doxycycline. In this set of studies, analysis of RoI from the basolateral surface quantified the hM_1_-mEGFP receptor construct to be present at 41.8 ± 1.3 copies·μm^−2^ (*n* = 134) with a median MEU of 1.12 (Fig. 7*A*). SpIDA indicated that in 26.9% of the RoI receptors were present dominantly within dimeric/oligomeric complexes. Treatment of the cells with pirenzepine (10 μm) for 90 min did result in a small increase (*p* < 0.01) in overall basolateral receptor density to 54.1 ± 1.7 copies·μm^−2^ (*n* = 134) (Fig. 7*A*). However, whether including the full data set (median QB after pirenzepine, 1.47 times MEU) or only a subset of the data in which observations at receptor density levels of >80 copies·μm^−2^ were excluded from the analysis, 63.4% of the RoIs now reflected receptors dominantly in dimer/oligomer forms, a major (*p* < 0.001) increase over the untreated cells (Fig. 7*A*). To assess the effect of pirenzepine further, we examined the reversibility of the ligand-induced dimerization/oligomerization. Following treatment with pirenzepine for 90 min, the cells were washed to remove ligand from the medium, and then the degree of receptor complexity was assessed 90 min later to allow time for bound pirenzepine to dissociate from the receptors. This resulted in a dramatic reversal in the pattern of receptor complexity (Fig. 7*B*) with now only 16.2% of the hM_1_-mEGFP RoIs being defined as dimeric/oligomeric, a percentage that was not different (*p* > 0.05) from that observed in sets of untreated cells that were analyzed in parallel (Fig. 7*B*).

**FIGURE 8.**
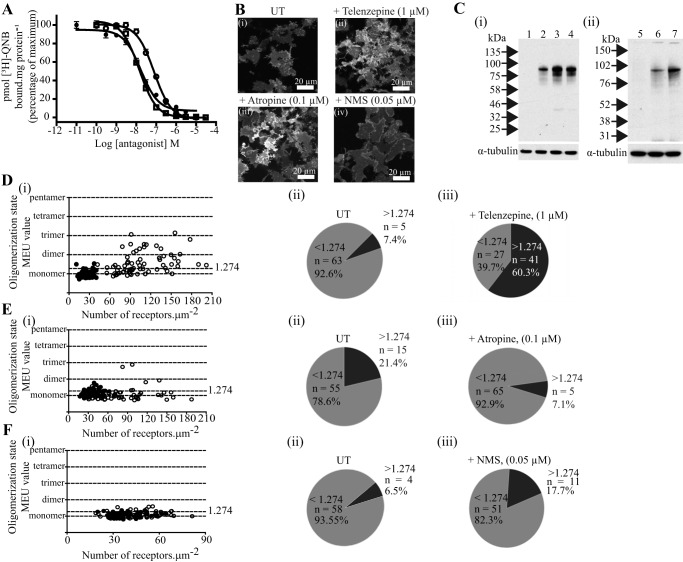
**Telenzepine, but not atropine or *N*-methylscopolamine, also promotes M_1_ receptor oligomerization.** As in Fig. 3, cells harboring hM_1_-mEGFP were treated with 100 ng·ml^−1^ doxycycline for 24 h to induce expression of the receptor construct. *A*, membrane preparations from these cells were used in ligand binding studies using a fixed concentration of [^3^H]QNB (1 nm) and varying concentrations of either telenzepine (*open circles*), atropine (*open squares*), or *N*-methylscopolamine (*filled circles*). Shown are representative plots from *n* = 3; *error bars* represent ±S.E. *B*, images of the basolateral surface of cells treated with vehicle (*UT*) (*panel i*), telenzepine (*panel ii*), atropine (*panel iii*), or NMS (*panel iv*). *C*, immunoblotting of cell lysates confirmed up-regulation of hM_1_-mEGFP following treatment with telenzepine (*panel i*, *lane 3*), atropine (*panel i*, *lane 4*), or *N*-methylscopolamine (*panel ii*, *lane 7*). Cell lysate from uninduced cells (*panel i*, *lane 1* and *panel ii*, *lane 5*) and from cells treated with the vehicle (*panel i*, *lane 2* and *panel ii*, *lane 6*) were also loaded for comparison. Detection of α-tubulin acted as a loading control. *D*, *E*, and *F*, comparisons of QB (presented as monomeric equivalent units) and receptor expression levels in cells induced to express hM_1_-mEGFP by treatment with 100 ng·ml^−1^ doxycycline that were then untreated (*filled symbols*) or treated with 1 μm telenzepine (*D*, *panel i*, *open symbols*), 0.1 μm atropine (*E*, *panel i*, *open symbols*), or 0.05 μm *N*-methylscopolamine (F, *panel i*, *open symbols*) for 16 h. Although treatment with telenzepine produced a large increase in the proportion of RoIs containing predominantly receptor dimers/oligomers (*D*, *panels ii* and *iii*), atropine (*E*, *panels ii* and *iii*), and *N*-methylscopolamine (*F*, *panels ii* and *iii*) did not.

Telenzepine is structurally closely related to pirenzepine (Fig. 1) and is also an M_1_-selective antagonist. Competition binding studies using [^3^H]QNB and telenzepine indicated that telenzepine (p*K_i_* = 8.33 ± 0.04) displayed some 4-fold higher affinity than pirenzepine (Fig. 8*A*). Telenzepine is also known to dissociate slowly from the M_1_ receptor after binding. This feature allowed fluorophore-labeled forms of telenzepine to be used to monitor the distribution and movement of molecules of the M_1_ receptor expressed in CHO cells (7). Overnight treatment with telenzepine (1 μm) of Flp-In T-REx 293 cells induced to express hM_1_-mEGFP by treatment with 100 ng·ml^−1^ doxycycline also produced a very marked up-regulation (*p* < 0.001) of levels of the receptor as detected by both imaging the basolateral surface of the cells (Fig. 8*B*, *panels i* and *ii*) and immunoblotting studies (Fig. 8*C*). Treatment with telenzepine also enhanced quaternary structure complexity of the receptor (Fig. 8*D*) in this specific set of experiments from a very modest basal level of 7.4% of RoIs that was consistent with the receptor being in dimeric/oligomeric complexes to 60.3%. We also assessed whether an equivalent effect was produced by a more traditional, non-subtype selective muscarinic antagonist. Atropine is the prototypic muscarinic blocker and, based on competition with [^3^H]QNB to bind to the M_1_ receptor construct (Fig. 8*A*), has high affinity for this receptor (p*K_i_* = 9.06 ± 0.04). Sustained treatment of cells induced to express hM_1_-mEGFP with atropine (100 nm) also produced a significant (*p* < 0.05) up-regulation of the amount of the receptor construct at the basolateral surface of these cells from 31.7 ± 1.0 to 74.4 ± 4.6 receptors·μm^−2^ (Fig. 8*B*, *panel iii*), an effect that was also detected in immunoblotting studies (Fig. 8*C*). However, analysis by SpIDA showed that, in contrast with pirenzepine and telenzepine, atropine treatment did not result in an overall increase in the proportion of RoIs assessed as containing predominantly receptor dimers (Fig. 8*E*), although observation of individual RoI did suggest the presence of a small proportion of higher complexes (Fig. 8*E*). Based on this potentially surprising difference, we also explored the effects of a further muscarinic receptor subtype non-selective antagonist. We selected NMS because it is structurally related to atropine. Competition binding studies with [^3^H]QNB indicated NMS to bind hM_1_-mEGFP with high affinity (p*K_i_* = 9.16 ± 0.17 (Fig. 8*A*). Sustained treatment of cells with NMS (50 nm) again produced a degree of up-regulation of hM_1_-mEGFP levels as assessed by immunoblotting studies (Fig. 8*C*). However, as both imaging studies of the basolateral membrane (Fig. 8*B*, *panel iv*) and quantification of fluorescence intensity of imaged RoIs (Fig. 8*F*) did not demonstrate this effect, it is assumed the extra copies of the receptor detected by the immunoblotting reflect that the cellular location of these are not within the basolateral membrane. Notably, as for atropine, sustained treatment with NMS had very limited effect on hM_1_-mEGFP quaternary organization (Fig. 8*F*).

**FIGURE 9.**
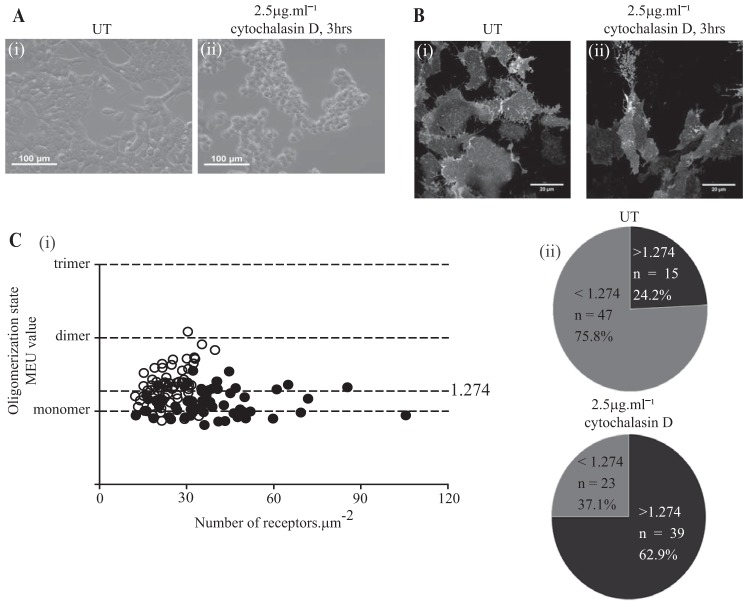
**Actin cytoskeleton destabilization promotes M_1_ receptor oligomerization.** Flp-In T-REx 293 cells harboring hM_1_-mEGFP were treated with doxycycline (100 ng·ml^−1^) for 24 h. *A*, bright field images of cells treated with vehicle (*UT*) (*panel i*) or with cytochalasin D (*panel ii*). *B*, images of the basolateral surface of cells treated with vehicle (*UT*) (*panel i*) or cytochalasin D (*panel ii*). *C*, comparisons of QB (presented as monomeric equivalent units) and receptor expression levels in cells treated with the vehicle (*panel i*, *filled symbols*) or with cytochalasin D (*panel i*, *open symbols*) for 3 h. Treatment with cytochalasin D produced a substantial increase in the proportion of RoIs containing a high percentage of receptor dimers/oligomers (*panel ii*).

The contribution of cytoskeletal structure to hM_1_-mEGFP quaternary organization was then assessed by treatment of cells induced to express hM_1_-mEGFP with the actin-depolymerizing agent cytochalasin D (2.5 μg·ml^−1^; 3 h). This treatment generated a number of challenges for SpIDA. As anticipated, this treatment resulted in the cells becoming more rounded (Fig. 9*A*) and, as anticipated from this, having reduced basolateral membrane contact with the glass coverslip (Fig. 9*B*). However, following control measurements in cells induced to express the P-M-mEGFP construct and similarly treated with cytochalasin D, we were able to perform SpIDA studies (Fig. 9*C*). Treatment with cytochalasin D for this period did not up-regulate basolateral levels of hM_1_-mEGFP (Fig. 9*C*) but did increase receptor quaternary structure complexity (Fig. 9*C*). Interestingly, using a very different approach, a similar observation has been made for the serotonin 5-HT_1A_ receptor (28).

**FIGURE 10.**
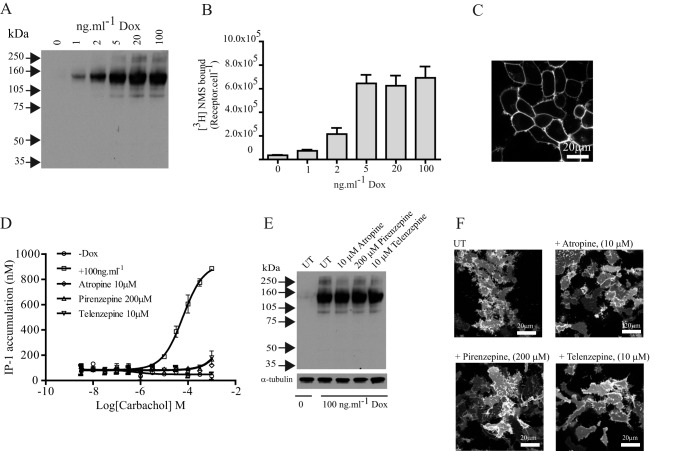
**Muscarinic antagonists do not up-regulate levels of the M_3_ receptor.** Flp-In T-REx 293 cells harboring hM_3_-mEGFP were treated with the indicated concentrations of doxycycline (*Dox*) for 24 h to induce expression of the receptor construct. *A*, anti-GFP immunoblotting of cell lysates detected hM_3_-mEGFP as a 140-kDa polypeptide. *B*, specific binding of [^3^H]NMS confirmed that the level of expression of hM_3_-mEGFP at the surface of intact cells is dependent on doxycycline concentration. Shown are combined data from *n* = 3 experiments; *error bars* represent ±S.E. *C*, confocal image of cells induced to express hM_3_-mEGFP shows it to be restricted to the plasma membrane. *D*, the ability of the muscarinic agonist carbachol to promote generation of inositol monophosphates in these cells is dependent upon treatment with doxycycline (compare −*Dox* and 100 ng·ml^−1^) and is fully blocked by the indicated concentrations of each of atropine, pirenzepine, and telenzepine. Shown are representative plots from *n* = 3; *error bars* represent ±S.E. *E* and *F*, sustained treatment with atropine, pirenzepine, or telenzepine does not up-regulate levels of hM_3_-mEGFP. *E*, anti-GFP immunoblotting was performed on SDS-PAGE-resolved lysates from cells not induced with doxycycline or induced to express hM_3_-mEGFP and then treated for 16 h with the indicated concentration of atropine, pirenzepine, or telenzepine. Detection of α-tubulin acted as a loading control. *F*, images of the basolateral surface of such cells. *Scale bar*, 20 μm. *UT*, untreated.

Although pirenzepine and telenzepine are M_1_-selective antagonists, they also can interact with other muscarinic receptor subtypes. The M_3_ receptor is closely related to the M_1_ subtype and also signals selectively via G_q_/G_11_ family G proteins. An M_3_ muscarinic receptor construct (hM_3_-mEGFP) akin to hM_1_-mEGFP was generated and cloned into the Flp-In T-REx locus of Flp-In T-REx 293 cells to allow doxycycline-controlled regulation of expression (Fig. 10*A*). The overall size of the M_3_ receptor is considerably larger than the M_1_ subtype, largely because of a markedly longer third intracellular loop. As such, the apparent molecular mass of this construct (approximately 140 kDa) was much greater in anti-GFP immunoblots of SDS-PAGE-resolved lysates of these cells (Fig. 10*A*). [^3^H]NMS is a high affinity muscarinic antagonist that, because it is hydrophilic and, therefore, does not easily cross the plasma membrane of intact cells, can be used to identify cell surface muscarinic receptors. Specific binding of a near saturating concentration of this ligand also confirmed doxycycline concentration-dependent expression of hM_3_-mEGFP (Fig. 10*B*). Confocal imaging of cells induced to express hM_3_-mEGFP indicated that most of the receptor was present at the plasma membrane, in this case even without treatment with an antagonist ligand (Fig. 10*C*). As anticipated for a G_q_/G_11_-coupled receptor, the muscarinic agonist carbachol stimulated production of inositol phosphates in a concentration-dependent fashion in cells induced to express hM_3_-mEGFP but not in cells that were not pretreated with doxycycline (Fig. 10*D*). Competition binding studies using [^3^H]QNB (*K_d_* for hM_3_-mEGFP = 0.28 ± 0.05 nm) allowed estimation of the affinity of pirenzepine (p*K_i_* = 5.92 ± 0.09), telenzepine (p*K_i_* = 7.15 ± 0.02), and atropine (p*K_i_* = 7.97 ± 0.06). Importantly, at concentrations assessed from [^3^H]QNB competition binding experiments to be at least 100 times *K_i_*, each of pirenzepine (200 μm), telenzepine (10 μm), and atropine (10 μm) fully blocked carbachol-induced inositol phosphate generation (Fig. 10*D*). Notably, unlike the hM_1_-mEGFP construct, sustained treatment with any of these three antagonists at these concentrations failed to up-regulate levels of hM_3_-mEGFP whether assessed via immunoblotting studies performed on cell lysates (Fig. 10*E*) or by direct observation of the basolateral surface of the cells (Fig. 10*F*).

**FIGURE 11.**
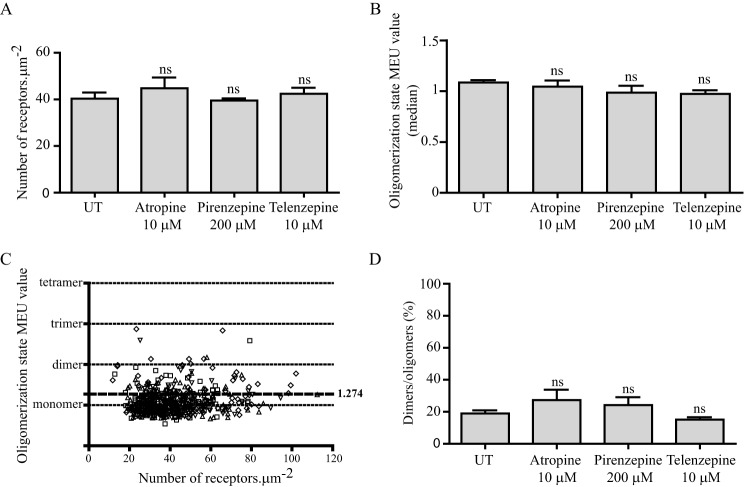
**Muscarinic antagonists do not affect the quaternary organization of the M_3_ receptor.** Cells, as in Fig. 9, that had been induced to express hM_3_-mEGFP were treated with the indicated concentrations of atropine, pirenzepine, or telenzepine for 16 h. Imaging of RoI within the basolateral surface indicated that treatment with the ligands did not alter the mean receptor expression level (*A*) or the overall organizational state of the receptor (*B*). Data are presented as monomeric equivalent units. *C*, full data set of individual QB values (again expressed as monomeric equivalent units) for untreated cells (*squares*) and those treated with atropine (*diamonds*), pirenzepine (*triangles*), or telenzepine (*inverted triangles*). *D*, assessment of percentage of RoI containing predominantly dimers/oligomers following each treatment. *ns*, not significantly different from untreated. All graphs show combined data from at least *n* = 3 experiments; *error bars* represent ±S.E. *UT*, untreated.

When these cells were induced to express hM_3_-mEGFP using a maximally effective concentration of doxycycline (100 ng·ml^−1^), expression at the basolateral surface was measured to be 40.3 ± 2.6 copies·μm^−2^ (Fig. 11*A*). The median QB corresponded to 1.08 MEU (*n* = 132) (Fig. 11*B*). SpIDA of the individual observations indicated that in 81.1% of the RoIs the receptor was predominantly monomeric. Analysis of RoIs following sustained treatment with each of pirenzepine, telenzepine, and atropine confirmed that these ligands did not alter the mean expression levels of hM_3_-mEGFP (Fig. 11*A*), and neither did they alter the QB observed (Fig. 11*B*). Moreover, analysis of individual observations did not support any regulation of the hM_3_ receptor monomer-oligomer distribution (Fig. 11, *C* and *D*).

## Discussion

Although it is well established that monomers of members of the rhodopsin-like, class A group of GPCRs can interact to generate dimers and/or higher order oligomers (1, 2), issues relating to the stability or otherwise of such interactions and how this might vary between both different receptors and in cells and tissues expressing different amounts of an individual receptor remain unresolved. Thus, although the formyl peptide receptor 1, when present in CHO cells at rather low expression levels, has been observed to interconvert between monomers and dimers with subsecond kinetics (29) and the hM_1_ receptor expressed in CHO cells has also been reported to exist as a rapidly interconverting mixture of monomers and dimers (7), the technical approaches used in these studies rely on and require low level expression. At the other extreme, concerns have been raised over the possibility that certain studies on the organizational structure of class A GPCRs have used high level receptor “overexpression” that might have produced outcomes in terms of receptor-receptor interactions that are not of physiological relevance (1, 2). However, it is important to note that, particularly in the CNS, ligand binding studies performed on membranes generated from bulk tissue have indicated that receptors such as the M_1_ muscarinic receptor are expressed, in striatum for example, at levels of at least 1 pmol·mg of membrane protein^−1^ (15), and this is presumably higher in cells that actually express the receptor, whereas other GPCRs, including cannabinoid and opioid receptors, are expressed regionally at significantly higher levels. It has also been suggested that receptor organizational complexity may increase with expression levels. Indeed, Calebiro *et al.* (30) observed just such a relationship for both the β_1_- and β_2_-adrenoceptors following expression in HEK293 cells and were able to detect increasing proportions of both dimers and oligomers of each receptor. Equally, a similar relationship between receptor expression level and quaternary complexity has also been observed for the serotonin 5-HT_2C_ receptor (24). As such, based solely on mass action, then in a number of natively expressing systems, GPCRs may potentially be at least partially dimeric and/or oligomeric. However, it is important to note that others have suggested more stable oligomeric organization over a significant range of expression levels. For example, Herrick-Davis *et al.* (5) have indicated that over a 10-fold range of expression, between 26,000 and 260,000 copies per cell, the M_1_ receptor expressed in HEK293 cells is consistently dimeric, and Guo *et al.* (31) suggested that over a 100-fold range of expression levels dopamine D_2_ receptors had cross-linking characteristics consistent with them forming and remaining as tetramers. This, however, is in contrast to other studies of the dopamine D_2_ receptor suggesting that “dimeric” interactions might, at best, be transient (32). Moreover, although at odds with earlier studies indicating that the serotonin 5-HT_2C_ receptor is also dimeric when expressed in HEK293 cells (33) and indeed is also reportedly dimeric in epithelial cells from choroid plexus where endogenous expression was estimated to be 32 receptors·μm^−2^ on the apical surface (34), Ward *et al.* (24) noted that at expression levels above 100 receptors·μm^−2^ on the basolateral surface of HEK293 cells much of the 5-HT_2C_ receptor was present within higher order oligomers rather than dimers and that at expression levels below 50 receptors·μm^−2^ a significant proportion of this receptor was monomeric.

Herein we note in the basal state and at moderate expression levels in HEK293 cells that, although the larger proportion of the hM_1_ receptor is monomeric, there is a clear fraction of dimeric forms. Moreover, treatment of the cells with the muscarinic M_1_ receptor-selective antagonist pirenzepine resulted in a substantial increase in the proportion of dimeric, as well as oligomeric, forms. This is interesting from three distinct perspectives. First, these results are in agreement with those of Ilien *et al.* (26), who, by using two-photon fluorescence correlation spectroscopy in cells expressing an N-terminally fluorescent protein-tagged variant of the hM_1_ receptor, were able to observe a rapid, pirenzepine-induced transition from a situation in which the receptor was predominantly monomeric to a mixture of monomers and dimers. Second, although sustained treatment of cells with pirenzepine produced a marked up-regulation of the basolateral levels of hM_1_ receptor that might have confounded interpretation of the effect of the antagonist, this concern was overcome by having the receptor expressed from a doxycycline-regulated locus whereby we were able to define conditions in which the number of receptors after sustained treatment with pirenzepine was not different from vehicle-treated cells. In this situation, there was also clearly a much greater proportion of hM_1_ dimers detected after pirenzepine treatment. This demonstrated explicitly that although pirenzepine treatment does up-regulate levels of the receptor, which inherently increases the proportion of receptor dimers/oligomers, it also stabilizes receptor dimers in a manner-independent from this. It is also important to note that for the P-M-mEGFP construct, at levels well above those used to study the muscarinic receptors, this polypeptide was identified exclusively as being monomeric. Therefore, simple aspects of proximity at the levels of expression used for the hM_1_ did not result in artifactual identification of apparently dimeric species. Third and perhaps most intriguingly, pirenzepine is structurally very closely related to telenzepine. A fluorophore-tagged form of telenzepine was used by Hern *et al.* (7) to label M_1_ receptors for total internal reflection microscopy studies that allowed tracking and analysis of receptor monomers and dimers. As we note herein, as for the fluorescent form of telenzepine, which was used at high receptor occupancy by Hern *et al.* (7), native telenzepine caused both substantial up-regulation of the hM_1_ receptor and promoted the presence of dimers. As such it is likely that Hern *et al.* (7) may have inadvertently generated a greater proportion of receptor dimers simply by their choice of fluorescent antagonist. A further fascinating feature of the studies with pirenzepine and telenzepine was that the effect of these ligands was not mimicked by the structurally distinct muscarinic antagonists atropine and NMS. Further studies will be required to unravel the molecular basis for these differences. One potential confounding issue for these studies would have been whether addition of pirenzepine and telenzepine altered the spectral characteristics of mEGFP. We tested this directly and observed no effect.

A further key point from these studies is that the effect of pirenzepine was reversible. Following removal of the ligand, the M_1_ receptor population returned to being predominantly monomeric. Although this is clearly the case, it was not realistic to accurately define the time course of this process. This reflects that, as a relatively high affinity antagonist for the hM_1_ receptor, pirenzepine has a significant residency time on the receptor. As such, simply washing cells to remove bulk ligand from the medium does not intrinsically remove the ligand from the receptor. Indeed, the slow dissociation rate of telenzepine from the M_1_ receptor was a key reason for Hern *et al.* (7) to select a fluorescent variant of telenzepine for their receptor tracking studies. Identification of a ligand that mimics the capacity of pirenzepine to promote M_1_ receptor dimerization but has rapid “off”-rate binding kinetics will be required, therefore, to start to define the true rate of hysteresis of M_1_ receptor quaternary complexes back to monomers in the absence of ligand.

Integral to the studies we have performed, SpIDA has been shown previously to be able to observe and quantify ligand-induced alterations in quaternary structure and organization of various classes of transmembrane receptor proteins. For example, using this method, we have shown that addition of epidermal growth factor results in rapid and ligand concentration-dependent dimerization of the epidermal growth factor receptor (24). Moreover, although SpIDA demonstrated the single transmembrane domain axonal guidance receptor Robo-1 to exist basally as a dimer and that this organization was unaffected by addition of the ligand Slit-2 (25), in terms of GPCRs, higher order oligomers and even dimers of the serotonin 5-HT_2C_ receptor were pushed toward monomeric organization upon binding of antagonist ligands from two distinct chemotypes (24). Furthermore, washing of the cells to remove bulk ligand from the medium allowed a time-dependent hysteresis of quaternary complexity back toward the basal state (24). Interestingly, ligand effects on receptor quaternary organization are clearly rather selective. The M_3_ muscarinic receptor is closely related to the M_1_ receptor and can also bind pirenzepine although with somewhat lower affinity. Pirenzepine did not produce an equivalent effect on the M_3_ receptor, however, even when the ligand was present at concentrations sufficient to fully occupy the M_3_ receptor population. The molecular basis for this distinction will require many further studies, including potentially the generation of receptor point mutants and chimeras between these subtypes.

The current studies highlight that the quaternary organization of class A GPCRs can indeed be regulated by ligand binding. Given that pirenzepine and telenzepine enhance this complexity for the hM_1_ receptor, whereas a group of antagonists decreased complexity of the serotonin 5-HT_2C_ receptor (24), then it will be important to explore this issue for other GPCRs in a systematic manner to begin to understand the molecular basis for these observations. Although in certain GPCRs a single transmembrane domain appears to provide a key symmetrical interface for receptor dimerization, *e.g.* transmembrane domain IV of the secretin receptor (35), studies on many GPCRs have concluded that multiple regions contribute (8, 11, 36). This has resulted in suggestions that there may be multiple ways in which receptor dimers can form, and the stability of these distinct forms may well differ (8, 37, 38). Moreover, there are suggestions, based particularly on receptor cross-linking studies, that ligands with different functionalities, *e.g.* agonists *versus* inverse agonists (39), alter details of the organization of receptor-receptor interfaces. This might well be anticipated to modify the affinity of interaction between the individual protomers and, therefore, the balance of observed quaternary structures. Clearly this is speculative, but as various mutations also alter the effectiveness of receptor dimerization and oligomerization (35–36) it would be likely that ligand effects at such mutants might be further amplified. These topics will form the basis of future studies.

## Author Contributions

G. M. conceived and coordinated the study and, with the assistance of all others, wrote the paper. J. D. P. and R. J. W. designed, performed, and analyzed the experiments shown in Figs. 2–9. S. M. designed, performed, and analyzed the experiments shown in Figs. 1, 8, 10, and 11. A. G. G. provided key insights into the SpIDA. All authors reviewed the results and approved the final version of the manuscript.

## Footnotes

3 The abbreviations used are:

GPCR: G protein-coupled receptor
mEGFP: monomeric enhanced green fluorescent protein
MEU: monomeric equivalent unit
NMS: *N*-methylscopolamine
P-M: palmitoylation-myristoylation
QB: quantal brightness
QNB: quinuclidinylbenzilate
RoI: region of interest
SpIDA: spatial intensity distribution analysis
Bis-Tris: 2-[bis(2-hydroxyethyl)amino]-2-(hydroxymethyl)propane-1,3-diol
hM: human M.
